# The relation between crystal structure and the occurrence of quantum-rotor-induced polarization

**DOI:** 10.5194/mr-2-751-2021

**Published:** 2021-10-22

**Authors:** Corinna Dietrich, Julia Wissel, Oliver Lorenz, Arafat Hossain Khan, Marko Bertmer, Somayeh Khazaei, Daniel Sebastiani, Jörg Matysik

**Affiliations:** 1 Institut für Analytische Chemie, Universität Leipzig, Linnéstr. 3, 04103 Leipzig, Germany; 2 Felix-Bloch-Institut für Festkörperphysik, Universität Leipzig, Linnéstr. 5, 04103 Leipzig, Germany; 3 Institut für Chemie, Martin-Luther-Universität Halle-Wittenberg, Von-Danckelmann-Platz 4, 06120 Halle, Germany; 4 Bioanalytical Chemistry, Technische Universität Dresden, Bergstraße 66, Dresden, Germany

## Abstract

Among hyperpolarization techniques, quantum-rotor-induced polarization (QRIP), also known as the Haupt effect, is a peculiar one. It is, on the one hand, rather simple to apply by cooling and heating a sample. On the other hand, only the methyl groups of a few substances seem to allow for the effect, which strongly limits the applicability of QRIP. While it is known that a high tunnel frequency is required, the structural conditions for the effect to occur have not been exhaustively studied yet. Here we report on our efforts to heuristically recognize structural motifs in molecular crystals able to allow to produce QRIP.

## 1 Introduction

**Figure 1:**
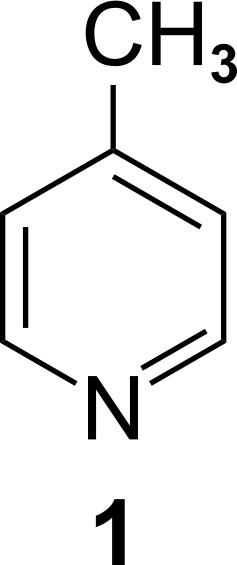
γ -picoline (**1**).

NMR spectroscopy is a very versatile analytical method; however, as caused by the low Boltzmann ratio, it suffers from a lack of sensitivity. Therefore, hyperpolarization methods are presently a hot topic (Halse, 2016; Köckenberger and Matysik, 2010; Kovtunov et al., 2018; Wang et al., 2019). Examples of these techniques are dynamic nuclear polarization (Ardenkjaer-Larsen, 2016; Kjeldsen et al., 2018; Lilly Thankamony et al., 2017; Milani et al., 2015; Ni et al., 2013), spin-exchange optical pumping (Hollenbach et al., 2016; Meersmann and Brunner, 2015; Norquay et al., 2018; Walker, 2011), photochemically induced dynamic nuclear polarization (Bode et al., 2012; Kiryutin et al., 2012; Sosnovsky et al., 2019), and para-hydrogen-induced polarization (Duckett and Mewis, 2012; Kiryutin et al., 2017; Korchak et al., 2009). Another technique is quantum-rotor-induced polarization (QRIP; Dumez et al., 2017; Horsewill, 1999; Icker et al., 2013; Icker and Berger, 2012; Meier, 2018). It was first observed by Haupt in γ -picoline (**1**; Fig. 1) during rapid temperature jumps at very low temperatures (Haupt, 1972, 1973).

**Figure 2:**
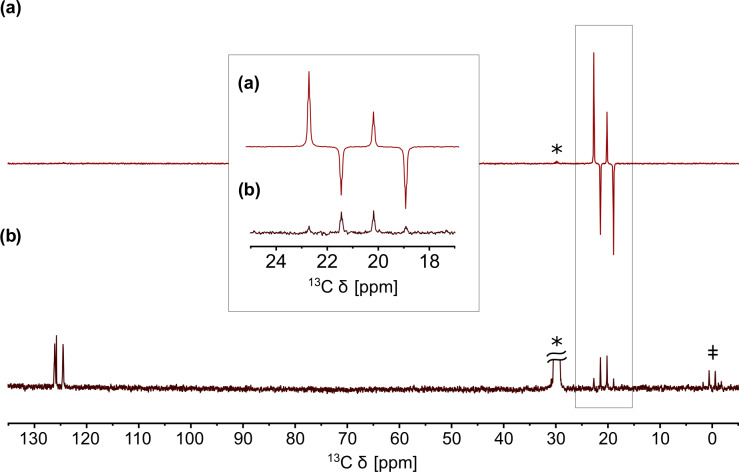
**(a)** QRIP-enhanced ${}^{13}C$ spectrum of γ -picoline (**1**) measured without proton decoupling, recorded with one scan after the cooling and dissolution procedure. Acetone-d6 was used as a solvent. **(b)** Reference spectrum recorded after full relaxation with 100 scans. The signals of acetone-d6 (labeled with an asterisk; ${}^{*}$ ) and tetramethylsilane (TMS; ‡ ) are clearly visible in the reference spectrum.

It is also possible to access the signal enhancement in a liquid state by freezing **1** at helium temperature, then rapidly dissolving it in deuterated solvents at room temperature, and measuring it immediately (Icker and Berger, 2012). With a custom-made setup, we were able to improve the safety and speed of the dissolution and transfer process, resulting in a higher signal enhancement factor of 530 (Dietrich et al., 2019). An example of a QRIP-enhanced spectrum is given in Fig. 2. The enhancement is limited to the signal of the methyl carbon and exhibits a hitherto unexpected antiphase pattern (Icker and Berger, 2012).

One might expect that more methyl-bearing compounds allow for QRIP, which would broaden the applicability of the effect. However, the work of Icker et al. (2013) indicated that only a few substances with methyl groups can be hyperpolarized in this way, and all of the positively tested compounds show a weaker hyperpolarization compared to **1**. It is also noted that QRIP is not limited to methyl groups and might occur in some other molecular rotors. While none of the other systems in Icker et al. (2013) showed signal enhancement, weak QRIP effects have been observed in the ${}^{17}O$ water–endofullerene complex (Meier et al., 2018). In the present study, we focus solely on the structural requirements for the occurrence of QRIP in methyl groups.

For a deeper understanding of these requirements, we discuss the underlying mechanisms of the effect. Thermodynamically, QRIP has been interpreted in terms of a resonant contact between a tunneling reservoir and a Zeeman reservoir (Horsewill, 1999) at low temperatures. The nuclear spin order is produced via coupling of the spin states to rotational quantum states of the methyl group. At the temperature of liquid helium, only the lowest rotational state is occupied. Upon a dramatic temperature jump to room temperature, a measurable non-Boltzmann distribution of nuclear spin states is gained via cross-relaxation effects. These relaxations also explain the antiphase pattern and are further described in Meier et al. (2013).

For QRIP, the tunnel splitting plays an important role. It is defined as the energy gap between the rotational ground state and the first excited state. The height of this difference determines the (low temperature) population ratio via the Boltzmann factor and, thus, the overall amplitude of the imposed spin symmetry constraint. Thus, it can be expected that high tunnel frequencies are strongly favorable for observing QRIP effects. In fact, **1** has an exceptionally high tunnel frequency of 520 µeV ( ∼4 cm ${}^{-1}$ ; Prager and Heidemann, 1997). The tunnel frequency is also linked to the capability of the methyl group to rotate freely (Barlow et al., 1992). Therefore, structural motifs with free methyl groups are especially interesting. In the case of **1**, the crystal structure shows a rather special feature. Each methyl group is paired with another one, and the pairs are all aligned perfectly in a face-to-face manner. Around the methyl pairs, the chemical environment creates rotational potential energy barriers (often with a C ${}_{3}$ or C ${}_{6}$ symmetry). There is a strong coupling of both these methyl groups (due to their spatial proximity) with a 2π/6 phase difference, which means that the superposition of the two rotational potential energy functions becomes surprisingly flat (i.e., the hills of the first rotational potential just fit to the valleys of the second potential function). This, in turn, leads to the possibility of the joint rotation of the methyl groups at a very low rotational barrier, which is virtually a free rotation, eventually resulting in a very high tunnel splitting (Khazaei and Sebastiani, 2017).

In the present work, we therefore search for substances which have one or several of these features, i.e., methyl groups with low steric hindrance, methyl groups in a similar distance to each other and a face-to-face arrangement as in **1**, and methyl groups with concerted rotations.

## 2 Materials and methods

### 2.1 Liquid-state NMR

Experiments were carried out on a Bruker Fourier 300 and a Bruker DRX 400 spectrometer. For the QRIP studies, samples were cooled for 90 min in liquid helium and subsequently mixed with deuterated solvents at room temperature. The mixture was transferred to the magnet and measured immediately. This procedure was carried out manually or with the self-built transfer system, where the mixing and the transfer of the solution into the magnet is carried out in one step during 35 s (Dietrich et al., 2019). If suitable, the transfer system has been preferred, due to faster sample transfer into the magnet. In cases of insufficient solubility, only the manual procedure has been found to be applicable. To validate structures and determine the signal enhancement factor, reference spectra were measured after full relaxation of the enhancement. Therefore, multiple scans were recorded, whereas QRIP enhanced spectra have been obtained with a single scan.

### 2.2 Solid-state NMR

For the solid-state experiments under magic angle spinning (MAS), a Bruker Avance III spectrometer (400 MHz ${}^{1}$ H frequency) was used. In order to test for QRIP enhancement, the powder sample was packed into a 4 mm zirconia rotor, closed with a zirconia cap, and cooled for 90 min in liquid helium (4.2 K). After cooling, the rotor was transferred manually into the magnet, and spectra were recorded at room temperature. For the measurement under vacuum, the powder sample was filled into a glass tube (3 mm outer diameter) and evacuated over 2 d. Afterwards, the glass tube was sealed and fitted into the 4 mm zirconia rotor with polytetrafluoroethylene stoppers (Khan et al., 2018). The rotor was closed with a zirconia cap and used as before. In every case, non-decoupled Hahn echo pulse sequences were used, and the spinning frequency was set to 8 kHz. Again, reference spectra with multiple scans were recorded afterwards.

### 2.3 Signal enhancement factor

To compare QRIP-enhanced spectra with one scan to reference spectra with multiple scans, the enhancement factor ε has been calculated by using Eq. (1). $(S/N{)}_{\mathrm{QRIP}}$ is the signal-to-noise ratio of the QRIP-enhanced signal, and $(S/N{)}_{\mathrm{ref}}$ is the signal-to-noise ratio of the reference spectrum with multiple scans. The number of scans is given as $n_{\mathrm{ref}}$ (Dietrich et al., 2019). S/N ratios were obtained from the Topspin 3.1 software.

1 $$\varepsilon =\frac{\sqrt{n_{\mathrm{ref}}}\cdot {\left(S/N\right)}_{\mathrm{QRIP}}}{{\left(S/N\right)}_{\mathrm{ref}}}.$$

### 2.4 Inelastic neutron scattering

Inelastic neutron scattering (INS) measurements were carried out with the time-of-flight–time-of-flight (TOF–TOF) instrument at the Forschungs-Neutronenquelle Heinz Maier-Leibnitz (Technical University of Munich, Garching, Germany; Lohstroh and Evenson, 2015).

### 2.5 X-ray diffraction

For the powder X-ray diffraction (PXRD) patterns, the samples were placed in 0.5 mm ∅ capillaries and measured using a STOE STADI P diffractometer (Cu K $\alpha_{1}$ radiation; equipped with a MYTHEN (Dectris) detector). Measurements were carried out at the Institute of Inorganic Chemistry, University of Leipzig.

### 2.6 Synthesis

γ -picoline hydrochloride (**2**) was commercially available (Carbosynth Limited), while γ -picoline nitrate (**3**) and γ –picoline hydrosulfate (**4**) were synthesized according to instructions from Wang et al. (2015) and Ullah et al. (2015).

## 3 Experimental results and discussion

In order to find clues for the structural requirements for the allowance or restriction of QRIP, a variety of compounds is investigated and the enhancement factor ε is obtained (Table 1).

**Table 1:** Overview of all substances which were experimentally or theoretically (crystal structure analysis) examined in this study. The reference numbers are bold to indicate a substance.

No.	Substance	QRIP ε	No.	Substance	QRIP ε
**1**	γ -picoline	60	**17**	Toluene@4- t -butylcalix[4]arene	NA
**2**	γ -picoline hydrochloride	–	**18**	Sodium acetate	low
**3**	γ -picoline nitrate	–	**19**	Acetonitrile	low
**4**	γ -picoline hydrosulfate	–	**20**	Acetone	low
**5**	Toluene	3	**21**	α -picoline	0
**6**	Lithium acetate dihydrate	20	**22**	p -xylene	0
**7**	N -( p -tolyl)acetamide	–	**23**	p -cresol	0
**8**	2,5-dimethyl-1,3-dinitrobenzene	–	**24**	m -cresol	low
**9**	N-(*tert*-butyl)acetamide	–	**25**	1,3-dibromo-2,4,6-trimethylbenzene	28
**10**	Ethyl carbamate	–	**26**	2-methoxynaphthalene	0
**11**	2-nitropropane	–	**27**	2,6-di- t -butylnaphthalene	0
**12**	$N^{\prime }$ -(3,4-difluorobenzylidene)-4-methylbenzenesulfonohydrazide	–	**28**	Cholesterol	0
**13**	acetylsalicylic acid	–		ZIF-8	–
**16**	Toluene@Calix[4]arene	NA		ZIF-67	–

NA – not available.

### 3.1 Chemical analogues of γ -picoline

**Figure 3:**
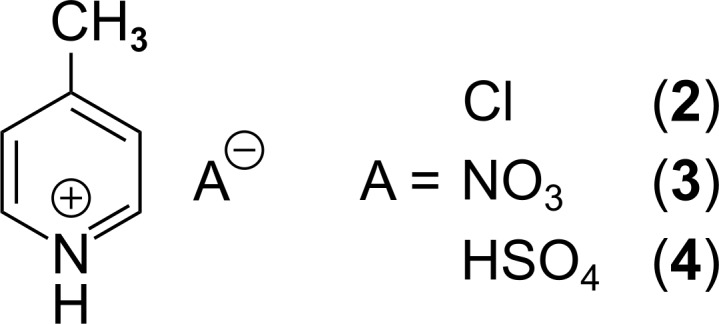
γ -picoline derivatives (**2**–**4**).

To gain a better understanding of the conditions for the occurrence of the effect, this heuristic study aims at finding connections between the various structural properties of a substance and the observed signal enhancement by QRIP. First, molecules that are similar to **1** in their molecular structure were searched and, as a result, very close analogues, i.e., three different salts of **1**, were found (Fig. 3).

All salts are solids at room temperature and are soluble in $H_{2}O$ . Hence, $D_{2}O$ was used as a solvent for the liquid-state NMR experiments. For the QRIP experiments, the manual transfer was chosen, since the high viscosity of $D_{2}O$ hinders the liquid flow in the transfer system and results in air bubbles in the NMR tube inside the magnet, which will lead to a disturbed signal. From each salt, 50 µmol were cooled for 90 min at 4.2 K and quickly dissolved in $D_{2}O$ ; the solution (inside the NMR tube) was shortly held in an ultrasonic bath to remove air bubbles and then transferred and measured. Even though the chemical structure and especially the chemical environment of the methyl group seems similar to **1**, no QRIP enhancement was observed for any of the three salts (**2** to **4**) in the ${}^{13}C$ NMR spectra. The ${}^{13}C$ NMR reference spectra are similar to the one of **1**, with slight chemical shift changes (see Table 2).

**Table 2:** ${}^{13}C$ NMR reference spectra of γ -picoline and its derivatives recorded on a Bruker Fourier 300 spectrometer. $D_{2}O$ was used as solvent. The chemical shifts are given in parts per million (ppm).

Substance	Assignment			
	**C**– ${\mathrm{CH}}_{3}$	N =**C**H–CH	CH–**C**H= $C_{q}$	**C**H ${}_{3}$
γ -picoline (**1**)	152.5	150.9	128.0	23.0
γ -picoline hydrochloride (**2**)	161.7	140.0	127.9	21.9
γ -picoline nitrate (**3**)	164.4	142.8	130.6	24.5
γ -picoline hydrosulfate (**4**)	164.5	142.8	130.7	24.5

Remarkably, also other chemical analogues, like the α -form and the β -form of picoline, show no signal enhancement as was shown in the work of Icker et al. (2013). They also studied toluene (**5**), which is, in its chemical structure, very similar to **1** and shows little QRIP enhancement. Hence, we conclude that not the molecular structure is decisive for the successful induction of QRIP and small modifications of the molecular structure can decide upon either QRIP induction or quenching.

**Figure 4:**
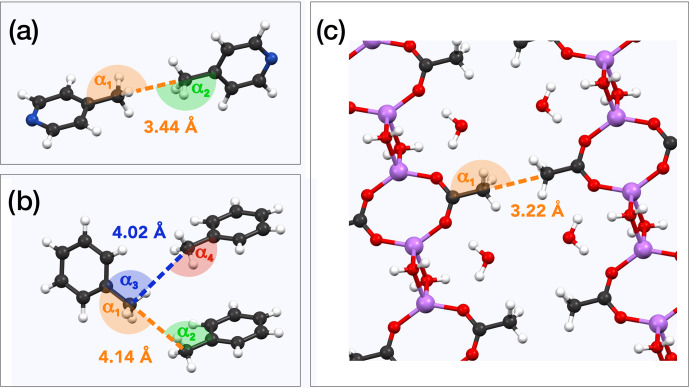
Examples for methyl pairs in the crystal structure. The distance between methyl pairs is given by the carbon-to-carbon distance. Angles are measured along the path from quaternary carbon via methyl carbon to the methyl carbon of the neighbor molecule. **(a)** γ -picoline (**1**) methyl pairs from crystal structure ZZZIVG, angles $\alpha_{1}$ and $\alpha_{2}$ , vary between 177 and 180 ${}^{\circ }$ (Ohms et al., 1985). **(b)** Toluene (**5**) methyl pairs (TOLUEN); the distance between the closest pairs is either 4.02 or 4.14 Å. Respective angles are $\alpha_{1}=165$ ${}^{\circ }$ , $\alpha_{2}=97$ ${}^{\circ }$ , $\alpha_{3}=94$ ${}^{\circ }$ , and $\alpha_{4}=157$ ${}^{\circ }$ (van der Putten et al., 1992). **(c)** Methyl pairs of lithium acetate dihydrate (**6**; LIACET); the angle $\alpha_{1}$ is 180 ${}^{\circ }$ (Galigné et al., 1970). The ionic bonds ( ${\mathrm{Ac}}^{-}\cdots {\mathrm{Li}}^{+}\cdots {\mathrm{OH}}_{2}$ ) are plotted the same as regular covalent bonds in order to improve the spatial comprehensibility of the crystal representation.

Searching for other parameters controlling the occurrence of QRIP, we recognize that lithium acetate dihydrate (**6**), which is no picoline analogue, shows moderate QRIP enhancement (weaker than **1**; stronger than **5**). Comparing the crystal structures, we found that both **1** and **6** exhibit pairs of methyl groups facing each other in a 180 ${}^{\circ }$ angle (Fig. 4a and c; see the crystal structures from Galigné et al., 1970, and Ohms et al., 1985), while the methyl groups in **5** have no such symmetry (Fig. 4b; see the crystal structure from van der Putten et al., 1992). This might explain the different tunnel frequencies, which directly affect QRIP (see Table 3; Icker et al., 2013; Meier et al., 2013). Since there is hardly empty space in the condensed phase, in most crystal structures the methyl groups cannot rotate freely. Only the direct compensation of two rotational barriers of two methyl groups which show a 180 ${}^{\circ }$ face-to-face arrangement allows for almost frictionless rotations of the coupled methyl pairs (concerted rotations; Khazaei and Sebastiani, 2017). Therefore, such a spatial arrangement in the crystal might provide a rare but well-defined structural feature allowing for induction of QRIP.

**Table 3:** Comparison of structural properties and QRIP. Methyl-methyl (Me-Me) distances were measured carbon to carbon, and angles between methyl groups were measured along the path from quaternary carbon via methyl carbon to the methyl carbon of the neighbor molecule (received from crystal structure data; Faber et al., 1999; Galigné et al., 1970; Ohms et al., 1985; van der Putten et al., 1992). Tunnel frequencies are from Prager and Heidemann (1997) and the QRIP signal enhancement factor ε from Icker et al. (2013). In addition to the name of the substance, the crystal structure code is given in parentheses.

Substance (structure code)	Me-Me distance	Me-Me angle	Tunnel frequency	QRIP ε
	(Å)	( ${}^{\circ }$ )	( µeV (cm ${}^{-1}$ ))	
γ -picoline (**1**; ZZZIVG)	3.44	177–180	520 ( ∼4 )	60
γ -picoline hydrochloride (**2**; DICCEX)	6.31	99	–	–
Toluene (**5**; TOLUEN)	4.02/4.14	94–165	28.5/26.0 ( ∼0.2 )	3
Lithium acetate dihydrate (**6**; LIACET)	3.22	180	250 ( ∼2 )	20

The present work aims to further corroborate the experimental evidence of this correlation. Hence, our next step has been the systematic search for substances which have structural properties similar to γ -picoline (**1**) in regard to the methyl-methyl distance and the face-to-face arrangement of the methyl groups. To this end, we searched for compounds of matching crystal structures.

### 3.2 Systematic crystal structure search

To find promising candidates for QRIP signal enhancement, the Cambridge Structural Database (CSD) was searched for substances with similar distances and angles between methyl groups compared to those values given in Table 3. Other desired properties were relatively small molecular size (to have a high methyl concentration and better chances to observe signal) and commercial availability. All six selected substances are listed in Table 4.

**Table 4:** Investigated compounds (**7**–**12**) from the systematic crystal structure search. Methyl-methyl (Me-Me) distances were measured carbon to carbon, and angles between methyl groups were measured along the path from quaternary carbon via methyl carbon to the methyl carbon of the neighbor molecule. The crystal structure data were obtained from the Cambridge Structural Database (CSD). In addition to the name of the substance, the crystal structure code is given in parentheses.

Substance (structure code)	Molecular structure	Me-Me	Me-Me
		distance	angle
		(Å)	( ${}^{\circ }$ )
N -( p -tolyl)acetamide	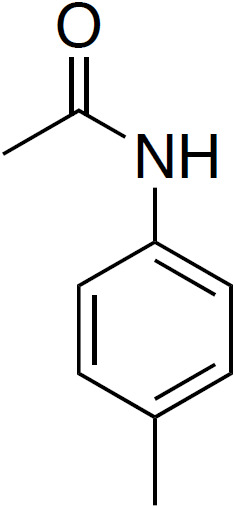	3.61	153/170
(**7**; ACTOLD)			
		
		
		
		
2,5-dimethyl-1,3-dinitrobenzene	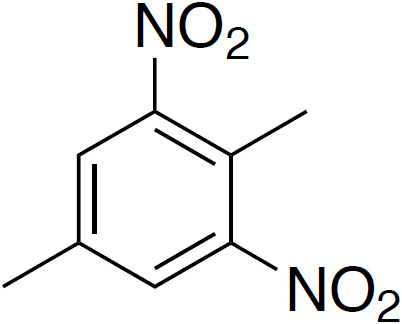	3.55	168
(**8**; AYOYAP)			
		
N-(*tert*-butyl)acetamide	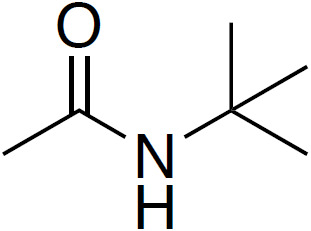	3.58	161
(**9**; APUYIU)			
		
Ethyl carbamate	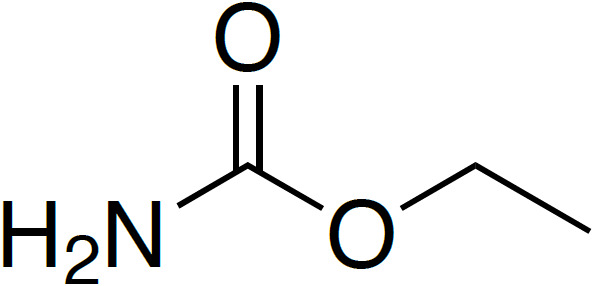	3.53	171
(**10**; ECARBM)			
		
2-nitropropane	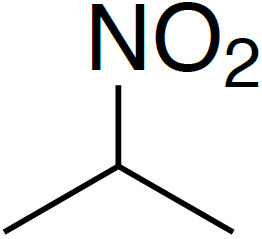	3.23/3.46	90–150
(**11**; IHIKIV)			
		
$N^{\prime }$ -(3,4-difluorobenzylidene)-4-methylbenzenesulfonohydrazide	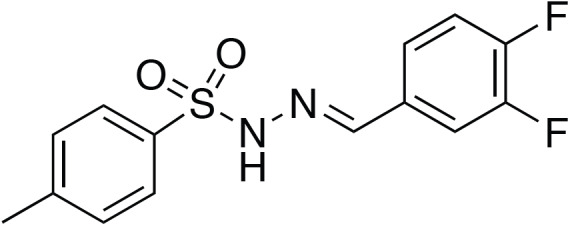	3.60	172
(**12**; NUQDUA)			
		
		

Although distances and angles between methyl groups of those compounds are in the range between compounds **1** and **5**, none of these compounds show a perfect face-to-face alignment of the methyl groups, and no QRIP enhancement was observed for any of them. The most probable reason is the occurrence of steric hindrance around the methyl groups in the crystal packing. This might affect the free rotation of the methyl groups and, thus, lead to lower tunnel frequencies inhibiting QRIP. Despite similar angles and distances of methyl groups, the impact of steric effects of the whole structure is difficult to estimate. To validate this correlation, theoretical calculations, as in Khazaei and Sebastiani (2016, 2017), and experimental measurements of tunneling frequencies are desirable. Another limitation can be a low concentration of methyl groups. In case of low QRIP (as exhibited in **5**), higher amounts of the sample were necessary to observe a QRIP-enhanced signal, i.e., 150 µmol for a good signal, whereas, for **1**, 50 µmol are sufficient to observe an intense QRIP-enhanced signal. For compounds **7** to **11**, depending on the solubility, between 50 and 100 µmol of the substance were used, and in the case of **12**, only 30 µmol were suitable.

**Figure 5:**
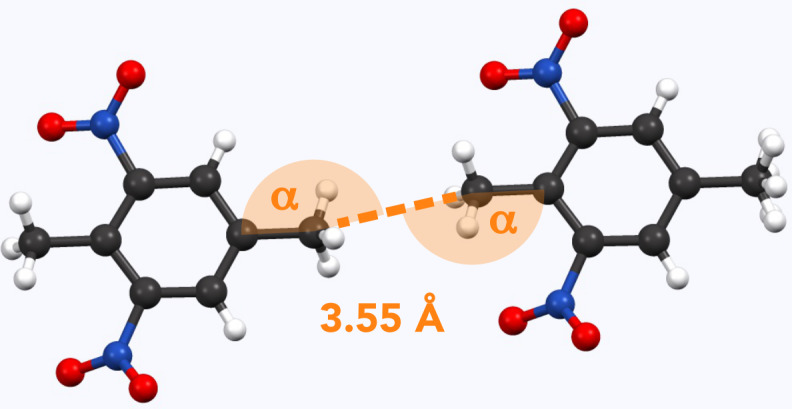
Example of methyl pairs in the crystal structure of **8** (AYOYAP; Johnston and Crather, 2011). The distance between them is 3.55 Å (measured carbon to carbon). Respective angles (measured carbon to carbon to carbon) are α=168 ${}^{\circ }$ .

**Figure 6:**
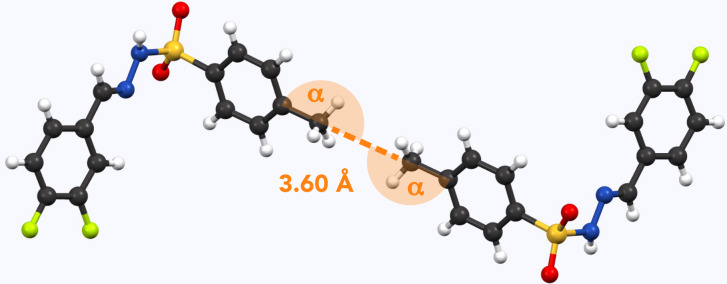
Example of methyl pairs in the crystal structure of **12** (NUQDUA; Wang and Yan, 2015). The distance between them is 3.60 Å (measured carbon to carbon). Respective angles (measured carbon to carbon to carbon) are α=172 ${}^{\circ }$ .

Compounds **8** and **12** were further investigated, since they were the two most promising candidates of this series regarding the methyl-methyl alignment. Interestingly, their methyl groups are in almost perfect face-to-face alignment (see Fig. 5; Johnston and Crather, 2011; see Fig. 6; Wang and Yan, 2015). On the other hand, they differ in the alignment of the attached phenyl rings compared to **1**. While the phenyl rings of two molecules lie in the same plane for **8** and **12**, they are tilted at 90 ${}^{\circ }$ to each other in the case of compound **1** (Fig. 4a). Whether this structural difference has an impact on QRIP requires further theoretical investigation. Since two dissolution experiments for **12** (30 µmol ) and three dissolution experiments for **8** (30–50 µmol ) showed no QRIP, we tried to figure out the reason for this result. To rule out the theory that the obtained substances are amorphous or possess another crystal structure as compared to the literature, we performed X-ray diffraction (XRD) and confirmed the correct crystal structure of compounds **8** and **12**.

Furthermore, the tunnel frequency has been investigated by inelastic neutron scattering (INS). A multi-peak fit allowed us to determine the first two transitions for each compound. For compound **8**, we determined values of 40±10 and 150±10 µeV (0.3 and 1.2 cm ${}^{-1}$ ). For compound **12**, we found 50±10 and 160±10 µeV (0.4 and 1.3 cm ${}^{-1}$ ). These values lie in the range between the tunnel frequencies of **5** and **6** (26 or 28 and 250 µeV ), which both exhibit QRIP enhancement but to a lower extent than **1**. Therefore, with regard to the tunnel frequencies, QRIP in compounds **8** and **12** is conceivable but very likely to exhibit only a weak enhancement.

It is noteworthy that, in **8**, each molecule possesses two methyl groups; thus, the methyl concentration is higher compared to tests on **1** and is not expected to be the limitation (30–50 µmol of **8** equals 60–100 µmol methyl groups). On the other hand, steric hindrance due to the NO ${}_{2}$ groups in close proximity to the methyl group might limit QRIP. In the case of **12**, there is no such hindrance through intramolecular factors; however, intermolecular hindrance is conceivable, and the low concentration (30 µmol ) is a probable limitation. Additionally, the size of the molecule can result in a faster decay of QRIP due to longer correlation times.

### 3.3 Aspirin

Next to the crystallographic databank approach, we searched for compounds having a particularly low frequency mode of the methyl group. In its crystal structure, aspirin (acetylsalicylic acid; **13**) has a particularly low frequency mode near 30 cm ${}^{-1}$ (3.7 meV), attributed to the concerted motions of methyl groups (Reilly and Tkatchenko, 2014). Compound **13** became an object of interest, since it exhibits some similarity to compound **1** in which the collective coupled motions of methyl groups are contributing to QRIP and the calculated methyl rotational barrier height of **1** is about 3.57 meV (Khazaei and Sebastiani, 2016). In analyzing the crystal structure of **13**, we found no face-to-face methyl pairs (ACSALA; Arputharaj et al., 2012). The closest methyl pairs are at a distance of 4.43 Å to each other, and the angles between them are 100 ${}^{\circ }$ /147 ${}^{\circ }$ . Multiple dissolution experiments showed no QRIP enhancement. According to Prager and Heidemann (1997), the tunnel frequency of **13** is 1.22 µeV (0.01 cm ${}^{-1}$ ), which is much lower than the tunnel frequencies of **1** and **2** (see Table 3). In fact, the mere coupling between two methyl groups (be it via a face-to-face arrangement or via lateral coupling similar to a cogwheel couple) is not sufficient for allowing a free rotation (leading to high tunnel splitting). A mandatory additional condition is that the rotational barriers created by the crystal surroundings have just the correct offset to each other. Assuming the common $C_{n}$ symmetries for the rotational barriers, this means that the maximum of the rotational potential for one of the methyl groups has to coincide exactly with the minimum of the rotational potential of the other one. This additional condition seems to not be fulfilled for aspirin, leading to the absence of QRIP enhancement.

### 3.4 Calixarene complexes

**Figure 7:**
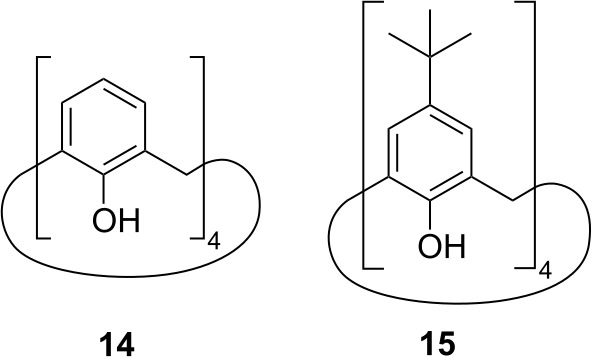
Structures of calix[4]arene (**14**) and 4-t-butylcalix[4]arene (**15**).

Furthermore, we considered two types of compounds, following our chemical intuition, namely calixarene compounds and metal organic frameworks. Calixarenes can occur in a cone shape and are therefore able to host smaller molecules like toluene (Gutsche et al., 1981). Because of the highly symmetric structure inside the calixarene cone, we suspected a favorable situation for the methyl group of the guest toluene molecule to rotate freely. Thus, there might be a possibility to observe QRIP enhancement in this complex. Hence, we tested two calixarenes as hosts, namely calix[4]arene (**14**) and 4- t -butylcalix[4]arene (**15**; see Fig. 7).

Complexes toluene@calix[4]arene (**16**) and toluene@4- t -butylcalix[4]arene (**17**) were synthesized by mixing a surplus of toluene with each calixarene at room temperature and letting the excess liquid dry (Andreetti et al., 1979).

In both cases, we did not succeed in obtaining a sufficiently high concentration in solution in order to perform QRIP experiments. This is due to the weak solubility of the calixarene complexes in CDCl ${}_{3}$ , acetone-d6, and toluene-d8 often resulting in opaque solutions or white suspensions with precipitate even for low concentrations. For compound **16**, the solubility is higher than for **17**. In the best case, we achieved an almost clear solution of 20 mg of **16** in CDCl ${}_{3}$ . Due to the higher mass of the complex in comparison to **1**, the resulting concentration is below what we expect to be observable by means of QRIP with the current setup. In future studies, calixarene complexes might be studied by solid-state NMR, avoiding the solubility issue. Furthermore, complexes with rather soluble calixarenes (Rehm et al., 2009) might provide an opportunity to induce hyperpolarization.

### 3.5 Metal organic frameworks (MOFs)

**Figure 8:**
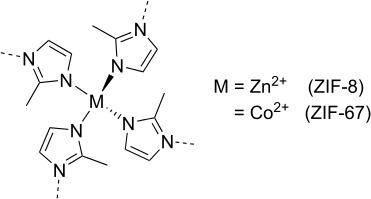
Structure of probed MOFs, i.e., ZIF-8 and ZIF-67.

Compared to molecular crystals, metal organic frameworks (MOFs) provide an alternative approach to observe freely rotating methyl groups. Methyl groups with low steric hindrance are, for example, expected in MOFs such as ZIF-8 and ZIF-67 (zeolitic imidazole framework; see Fig. 8). The difference in these two compounds lies in the different metal center atoms, i.e., Zn(II) in ZIF-8 and Co(II) in ZIF-67. Due to the specific structure allowing for pore formation, the methyl groups are pointing toward the center of these pores and, thus, can rotate freely, which has been shown at cryogenic temperatures (Li et al., 2018; Zhou et al.,  2008).

Due to the low solubility, and in order to avoid the hindrance of free rotating methyl groups by solvent molecules inside the pores, the measurements were carried out in solid-state NMR. To ensure that the carbon signal of both samples can be observed in general, reference spectra were recorded before and after the QRIP experiments. The assignments of the signals are given in Table 5.

**Table 5:** ${}^{13}C$ NMR reference spectra of ZIFs recorded on a Bruker Avance III spectrometer (400 MHz ${}^{1}$ H frequency; solid-state NMR). The chemical shifts are given in ppm.

Substance	Assignment		
	N–**C(**CH ${}_{3}{)}^{=}$ N	N–**C**H ${}^{=}$ **C**H–N	**C**H ${}_{3}$
ZIF-8	143.4	128-110	17-7
ZIF-67	143.3	130-110	17-7

In both cases, ${}^{13}C$ QRIP experiments were performed under MAS conditions. The resulting spectra showed no signal enhancement. A possible explanation for the absence of QRIP can be the adsorption of air molecules inside the pores of the ZIFs (Fairen-Jimenez et al., 2011), which might hinder the free rotation of the methyl groups. To exclude this, subsequent measurements under vacuum were performed. Also in this case, both ZIF samples did not show QRIP.

It is noteworthy that Co(II) is paramagnetic and, hence, signal broadening and otherwise unexpected chemical shifts (Bertini et al., 2005; Gueron, 1975; Vega and Fiat, 1976) were expected. However, the obtained reference spectrum of ZIF-67 shows no significant difference in the chemical shift in comparison to the Zn(II) analog. The signals are slightly broadened. Furthermore, an especially narrow line (20 Hz for ZIF-8 and 74 Hz for ZIF-67) at the far left of the spectrum is observed. The ${}^{13}C$ MAS NMR spectra were recorded without proton decoupling, since decoupling is expected to interfere with the QRIP effect. Therefore, CH and CH ${}_{3}$ signals of the ZIFs are broad, while the quaternary carbon is less affected. For the latter, the closest proton is the one from the methyl group, with a distance between the quaternary carbon and the methyl proton of 2.14 Å for ZIF-8 and 2.06 Å for ZIF-67. From this distance, we calculated a CH-dipole-dipole coupling of 3083 Hz (ZIF-8) and 3456 Hz (ZIF.67), which is averaged out at the chosen spinning speed of 8 kHz. With this and the high structural symmetry resulting in a low chemical shift anisotropy, the narrow line can be explained.

For both ZIFs, reference spectra with reasonable signal intensity were recorded after 1000 scans for the regular packing method and 10 000 scans for the advanced packing method with glass tubes. Following Eq. (1), the signal enhancement factor ε should be at least 32 (for regular packing or 100 for advanced packing) in order to observe signal with one scan. Smaller signal enhancement via QRIP is conceivable but could not be observed with the current setup. An intrinsic limitation to QRIP might be the proximity between the methyl groups inside the pores (5.0 Å in ZIF-8; crystal structure data from Morris et al., 2012; 4.6 Å in ZIF-76; Kwon et al., 2015; measured from carbon to carbon). This might also lead to the absence of signal. Considering the broad variety of MOFs, it is conceivable that some of them bear methyl groups which rotate more freely or undergo concerted rotations which are accessible for QRIP (Gangu et al., 2016; Gonzalez-Nelson et al., 2019; Kuc et al., 2007; Tarasi et al., 2020; Tian et al., 2007).

**Table 6:** List of compounds tested for QRIP in Icker et al. (2013) and Icker (2013). Methyl-methyl distances (measured carbon to carbon) and angles between methyl groups (measured along the path from quaternary carbon via methyl carbon to the methyl carbon of the neighbor molecule) were taken from the crystal structure data. Tunnel frequencies were taken from Prager and Heidemann (1997). In addition to the name of the substance, the crystal structure code is given in parentheses.

Substance (structure code)	Me-Me distance	Me-Me angle	Tunnel frequency	QRIP ε
	(Å)	( ${}^{\circ }$ )	( µeV (cm ${}^{-1}$ ))	
Sodium acetate (**18**; BOPKOG)	4.24	123/142	1.5 ( ∼0.01 )	Low
	3.45	90		
Acetonitrile (**19**; QQQCIV)	3.95	139	–	Low
Acetone (**20**; HIXHIF)	3.76	133/176	0.4 ( ∼0.003 )	Low
	3.91	132/158		
α -picoline (**21**; ZZZHKQ)	4.09	63/152	–	0
p -xylene (**22**; ZZZITY)	3.71	90/160	0.97 ( ∼0.008 )	0
	4.14	99		
α -picoline (**21**; ZZZHKQ)	4.09	63/152	–	0
p -xylene (**22**; ZZZITY)	3.71	90/160	0.97 ( ∼0.008 )	0
	4.14	99		
p -cresol (**23**; CRESOL)	4.01	95/113	–	0
m -cresol (**24**; MCRSOL)	3.99	79/176	–	Low
	4.06	174		
	3.89	83		
	3.94	83/114		
1,3-dibromo-2,4,6-trimethylbenzene	3.77	134	390 ( ∼3.1 )	28
(**25**; EJEROA)	3.78	142/158		
	4.08	129/156		
	3.74	80/83		
	4.03	99		
2-methoxynaphthalene (**26**; SAYRIT)	3.60	66/172	–	0
	4.05	73/107		
	4.21	125		
2,6-di- t -butylnaphthalene (**27**; KOKQUW)	3.75	155/161	–	0
	3.95	109/124		
	4.28	106/136		
Cholesterol (**28**; CHOEST)	4.37	98/138	–	0
	4.31	91/148		
	4.31	89/115		
	3.56	84/108		

### 3.6 Analysis of previous data

Aiming for heuristic data on the relation between structure and the hyperpolarization obtained by QRIP, we revisited the crystal structures of compounds studied in Icker et al. (2013) and Icker (2013). From all substances which were available in the CSD, angles and distances between methyl pairs were extracted. Here, we specifically searched for methyl pairs with a similar distance to that found in **1** (3.44 Å) and excluded all methyl pairs with a distance >4.4 Å. The results are given in Table 6. If available, the tunnel frequencies (Prager and Heidemann, 1997) were included in Table 6 as well. Interestingly, many methyl pairs with a distance in the range 3.45–4.37 Å were found, which is quite similar to **1** and makes methyl coupling conceivable. On the other hand, no other face-to-face methyl groups were found. Angles close to 180 ${}^{\circ }$ do not seem to be a sufficient argument to predict QRIP enhancement, as the comparison of two of the compounds shows; while **24** exhibits an 174 ${}^{\circ }$ angle at a methyl-methyl distance of 4.06 Å, it yields only a week polarization. Furthermore, a rather strong QRIP effect is observed in **25**, where the most promising methyl pair has similar distance (3.78 Å); however, at the same time, it is less aligned with angles of 142 ${}^{\circ }$ /158 ${}^{\circ }$ . The surprisingly high polarization in **25** is still below **1** but larger than in **6**, which is particularly curious since both **1** and **6** exhibit face-to-face methyl groups while **25** does not. Although the structure of **25** does not fit our assumptions to gain QRIP, the tunnel frequency is surprisingly high, which fits the presence of QRIP.

It is possible that the occurrence of multiple methyl groups in one molecule and multiple methyl pairs in the crystal structure are favorable for the likelihood of concerted rotations. However, those structural factors alone are also not sufficient for the prediction of QRIP, as other not polarizable or less polarizable substances like **7**–**9** (multiple methyl groups) contradict a general trend.

## 4 Conclusions

The aim of this study was to gain further understanding of the structural requirements of methyl bearing substances allowing for QRIP signal enhancement in NMR spectroscopy. Starting from the well-studied compound **1**, we found that its derivatives (**2**–**4**) do not exhibit QRIP. This indicates that structural similarity on a molecular level is insufficient for QRIP prediction. The weak polarization in **5** and absence of QRIP in α -picoline and β -picoline (Icker et al., 2013) supports this lack of correlation.

To better understand the specialty of **1**, we studied the crystal structure and recognized a rare structural feature; in its crystal structure, pairs of methyl groups are aligned in a perfect 180 ${}^{\circ }$ face-to-face manner. For the underlying tunnel effects, freely rotating methyl groups and high tunnel frequencies are favorable. Via concerted rotations, the face-to-face methyl groups in **1** can rotate exceptionally without friction, much like interacting gear wheels (Khazaei and Sebastiani, 2017; Meier et al., 2013).

In order to investigate the predictability and applicability of QRIP, we therefore searched for substances which show one or many of the aforementioned qualities, namely free rotation, promising alignment, high tunnel frequency of the methyl group, or concerted rotations of methyl groups. Thus, different approaches were tested.

First, we searched for compounds with similar methyl-methyl distances and angles as in **1** and found substances **7**–**12**. While all of them exhibit similar distances between methyl groups, they have no face-to-face arrangement of methyl groups and showed no QRIP enhancement. We conclude that either steric hindrance or missing positive interference of the methyl group is quenching the effect due to the less favorable arrangement.

Next, aspirin (**13**) was tested since it is described to have concerted motions of methyl groups. However, no QRIP enhancement was observed. We conclude that concerted rotations alone are insufficient. An additional condition is that the rotational barriers created by the crystal surroundings are just correctly offset from each other. This means that the maximum of the rotational potential for one of the methyl groups has to coincide exactly with the minimum of the rotational potential of the other one.

We further suspect that there are freely rotating methyl groups in complexes of toluene in calixarene cones and in MOFs. While the free rotation in calixarene complexes derives from a very symmetric surrounding of the methyl group, the MOFs show methyl groups in a relatively empty space. To this end, we did not succeed in performing QRIP measurements on calixarene complexes due to its low solubility. In MOFs, we did not observe QRIP enhancement.

Finally, we revisited previously studied compounds from Icker et al. (2013) and compared QRIP enhancement to methyl-methyl distances and angles. In the analyzed crystal structures of **18**–**28**, we found no face-to-face methyl groups but a variety of angles and distances between methyl pairs. However, no general trend or correlation between distances/angles and the enhancement factor was found. On the contrary, we found that **25** shows a higher polarization than **6**, despite the missing face-to-face arrangement. Although we were not able to recognize structural patterns in the crystal structures related to the appearance of QRIP, we confirm that a high tunnel barrier is required to induce QRIP.

To explain why promising candidates like **8** and **12** did not show QRIP and why **6** exhibits weaker QRIP than **1** (both show face-to-face methyl groups only with a slight difference in the methyl-methyl distance), we conclude that, similar to **13**, the necessary offset between rotational barriers of the methyl group is not given and, thus, QRIP is quenched. We also recognize that the mechanism of pairwise concerted rotation of two face-to-face methyl groups is not the only, and possibly not even the best, way to lower the rotational barrier of methyl rotation in a crystalline environment. Further candidates include a gear-like coupling of two adjacent methyl groups (which we did not, however, observe in any real molecule) and phonon modes of the molecular crystal, which could couple to the rotational motion of a methyl. Furthermore, cross relaxation is an essential part of QRIP and competing relaxation pathways would quench the effect. However, we did not focus on examining those effects in this work.

For further studies in this field, ${}^{13}C$ labeling can be a valid solution to finding weaker enhancement potentials in some of the studied, and possibly in other, compounds.

To summarize, we find with this study that even small structural differences can quench the QRIP effect by strongly affecting the tunnel frequency. Whether the crystal structure is a determining factor in general can neither be confirmed nor excluded. Therefore, we do not recognize a simple approach for predicting QRIP from structural assessment. Thus, a broader applicability of the effect on, for example, protein methyl groups is not to be expected.

## Supplement

10.5194/mr-2-751-2021-supplement The Supplement contains the following information: the NMR spectra of γ-picoline derivatives; crystal structure of γ-picoline hydrochloride (2), aspirin (13), m-cresol (24) and 1,3-dibromo-2,4,6-trimethylbenzene (25); pictures of the glass tubes for MAS NMR under air exclusion; XRD spectra and tunnel frequency fit of 2,5-dimethyl-1,3-dinitrobenzene (8) and N′-(3,4-difluorobenzylidene)-4-methylbenzenesulfonohydrazide (12). The supplement related to this article is available online at: https://doi.org/10.5194/mr-2-751-2021-supplement.

## Data Availability

NMR spectra were originally recorded with TopSpin and processed with MestreNova. The TopSpin files include the raw data and the pulse sequences. Those files and the XRD data (origin files) are available at https://doi.org/10.5281/zenodo.5078040 (Dietrich et al., 2021).
