# Supplementary material for: The relation between crystal structure and the occurrence of quantum-rotor-induced polarization
Source: Magn Reson (Gott). 2021 Oct 22;2(2):751–63. doi: 10.5194/mr-2-751-2021 (PMC10539751; doi:10.5194/mr-2-751-2021)
Supplement: The Supplement contains the following information: the NMR spectra of γ-picoline derivatives; crystal structure of γ-picoline hydrochloride (2), aspirin (13), m-cresol (24) and 1,3-dibromo-2,4,6-trimethylbenzene (25); pictures of the glass tubes for MAS NMR under air exclusion; XRD spectra and tunne [file mr-2-751-supplement.pdf]

Supplement of Magn. Reson., 2, 751–763, 2021
https://doi.org/10.5194/mr-2-751-2021-supplement
© Author(s) 2021. CC BY 4.0 License.

Open Access
MAGNETIC RESONANCE

Supplement of

The relation between crystal structure and the occurrence of quantum-rotor-induced polarization

Corinna Dietrich et al.

Correspondence to: Jörg Matysik (joerg.matysik@uni-leipzig.de) and Corinna Dietrich (corinna.dietrich@gmx.de)

The copyright of individual parts of the supplement might differ from the article licence.

NMR spectra of γ-picoline derivatives

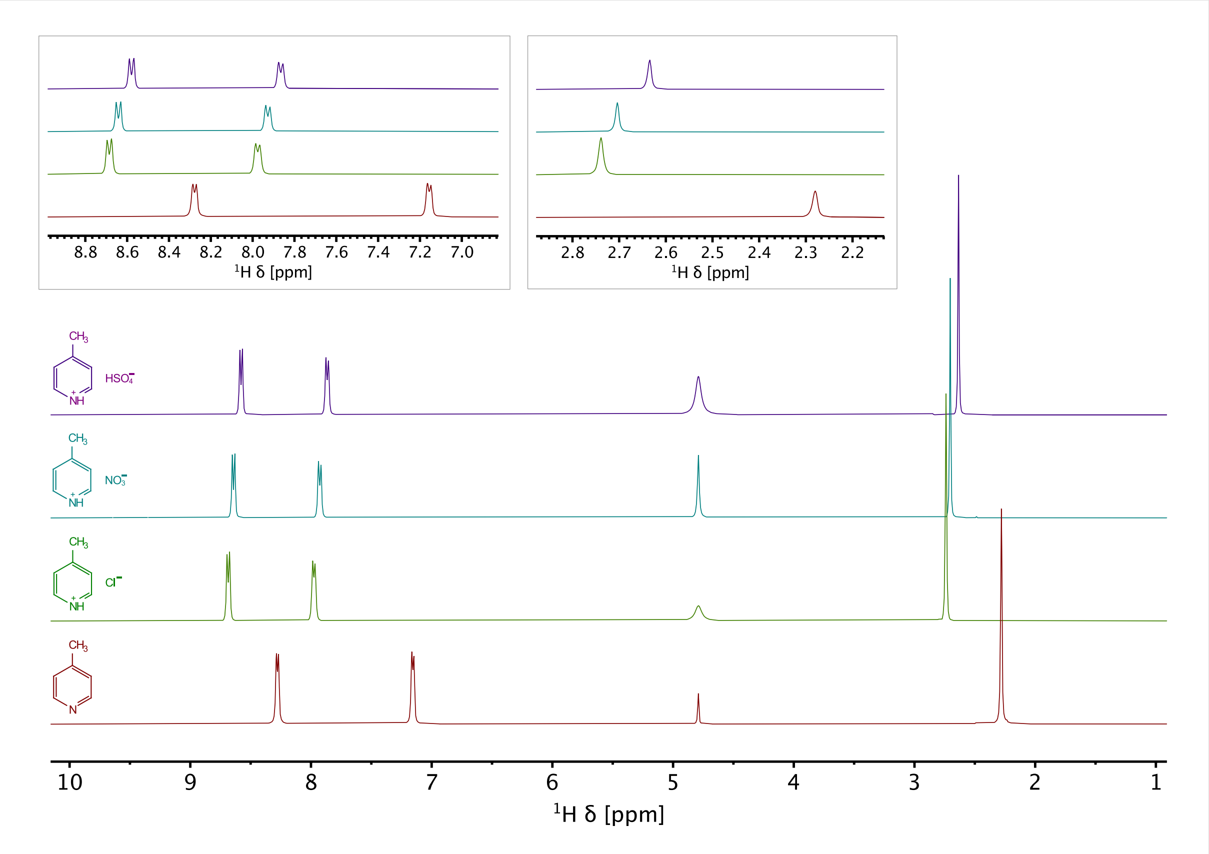

Fig. S1: Proton spectra of γ-picoline derivatives γ-picoline hydrochloride (**2**), γ-picoline nitrate (**3**) and γ-picoline hydrosulfate (**4**) in comparison to γ-picoline (**1**).

γ-picoline: δ (^{1}H, 300 MHz, D_2O) [ppm] = 8.28 (N---C**H**); 7.16 (CH---C**H**---C_q); 2.28 (C**H₃**).

γ-picoline hydrochloride: δ (^{1}H, 300 MHz, D_2O) [ppm] = 8.68 (N---C**H**); 7.98 (CH---C**H**---C_q); 2.74 (C**H₃**).
γ-picoline nitrate: δ (^{1}H, 300 MHz, D_2O) [ppm] = 8.64 (N---C**H**); 7.93 (CH---C**H**---C_q); 2.70 (C**H₃**).
γ-picoline hydrosulfate: δ (^{1}H, 300 MHz, D_2O) [ppm] = 8.58 (N---C**H**); 7.87 (CH---C**H**---C_q); 2.63 (C**H₃**).

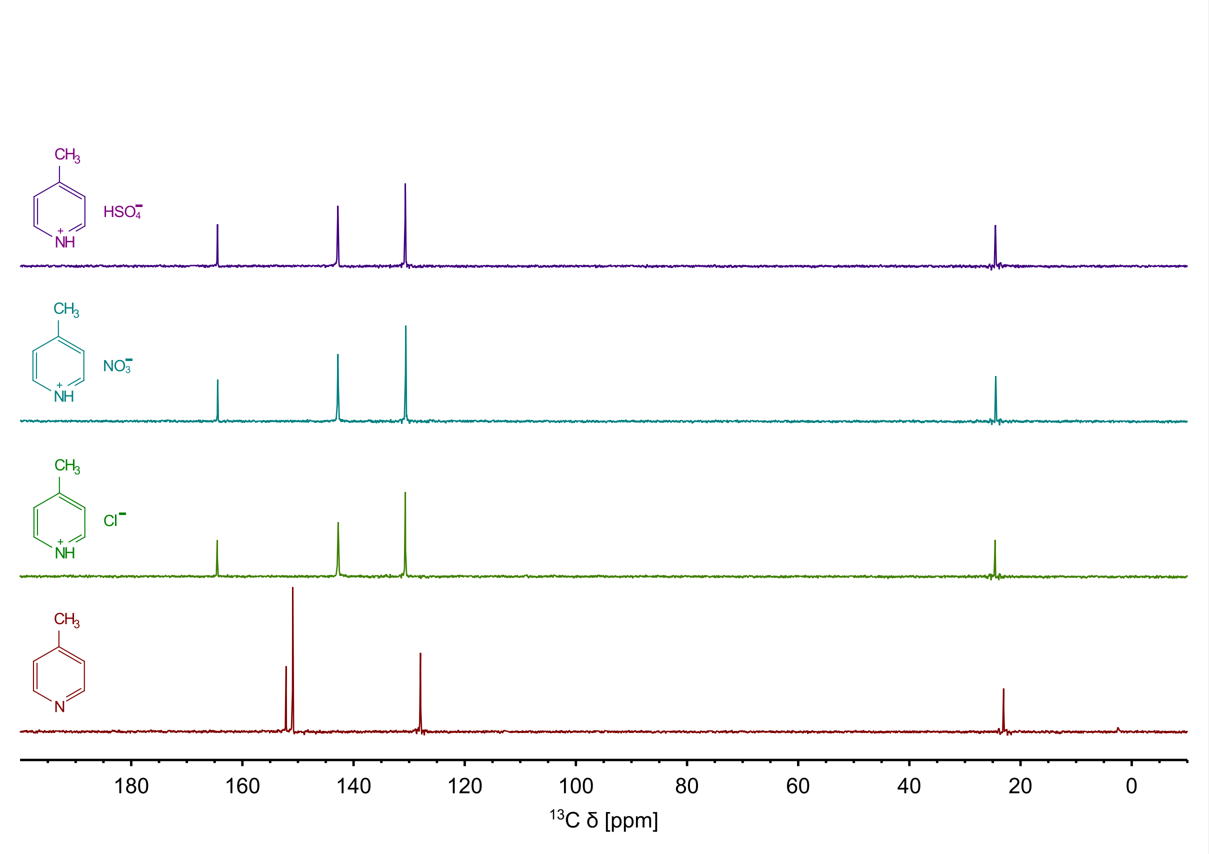

Fig. S2: ^{13}C spectra of γ-picoline derivatives **2**-**4**.

γ-picoline: δ (^{13}C, 300 MHz, D_2O) [ppm] = 152.5 (**C**---CH_3); 150.9 (N---**C**H---CH); 128.0 (CH---**C**H---C_q); 23.0 (**C**H_3).

γ-picoline hydrochloride: δ (^{13}C, 300 MHz, D_2O) [ppm] = 161.7 (**C**---CH_3); 140.0 (NH---**C**H---CH); 127.9 (CH---**C**H---C_q); 21.9 (**C**H_3).

γ-picoline nitrate: δ (^{13}C, 300 MHz, D_2O) [ppm] = 164.4 (**C**---CH_3); 142.8 (NH---**C**H---CH); 130.6 (CH---**C**H---C_q); 24.5 (**C**H_3).

γ-picoline hydrosulfate: δ (^{13}C, 300 MHz, D_2O) [ppm] = 164.5 (**C**---CH_3); 142.8 (NH---**C**H---CH); 130.7 (CH---**C**H---C_q); 24.5 (**C**H_3).

Molecular structure of γ-picoline hydrochloride

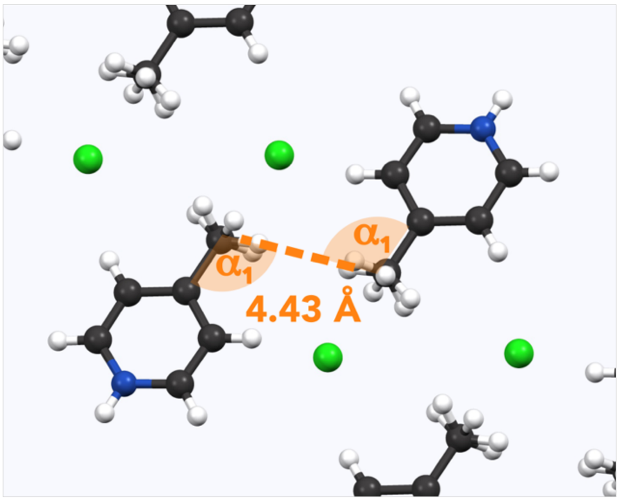

Fig. S3: Molecular structure of γ-picoline hydrochloride (**2**, DICCEX, [1]).

The angle α_1 is 103°. The molecular structures of **3** and **4** were not found in the Cambridge Crystallography Database.

Molecular structure of aspirin

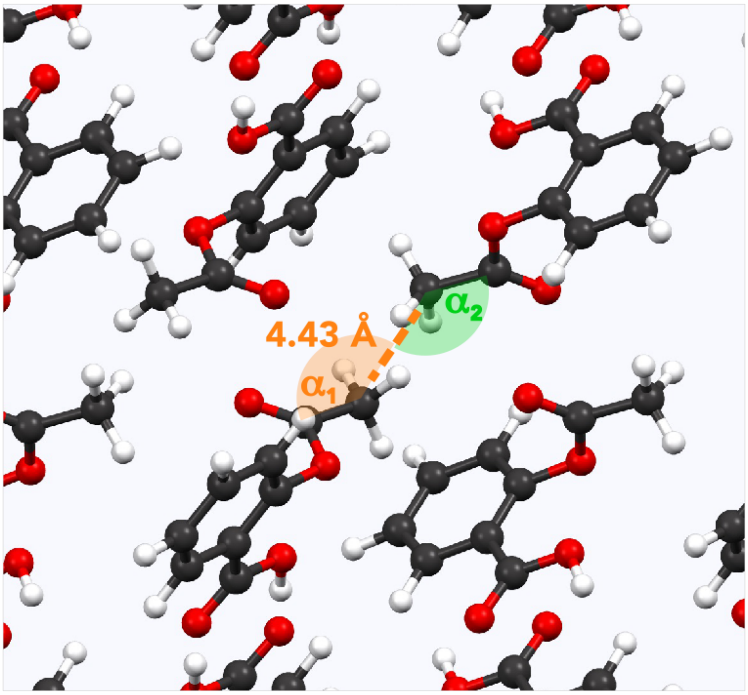

Fig. S4: Molecular structure of aspirin (**13**, ACSALA, [2]).

The angles are α_1 = 147° and α_2 = 100°.

Molecular structure of *m*-cresol

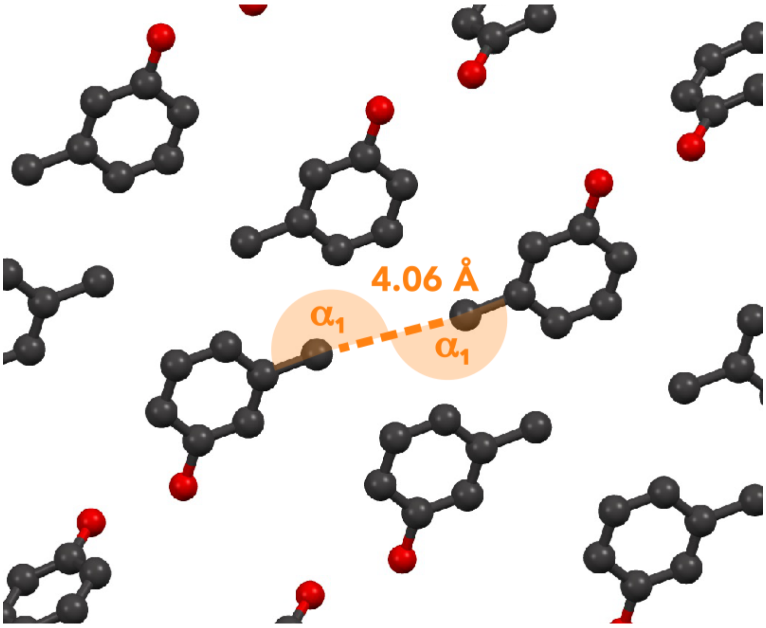

Fig. S5: Molecular structure of *m*-cresol (**24**, MCRSOL, [3]).

The angle α_1 is 174°.

Molecular structure of 1,3-dibromo-2,4,6-trimethylbenzene

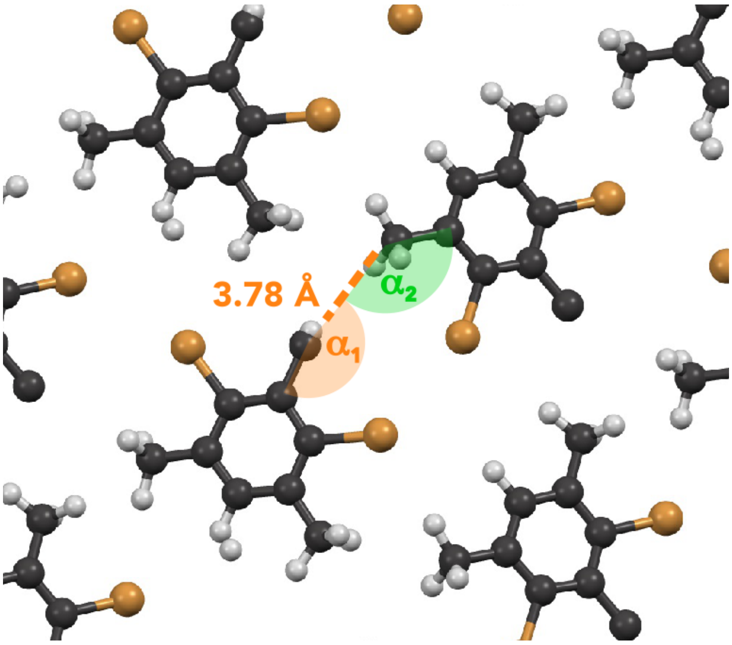

Fig. S6: Molecular structure of 1,3-dibromo-2,4,6-trimethylbenzene (**25**, EJEROA, [4]).

The angles are α_1 = 158° and α_2 = 142°.

Sample preparation for measurement under air exclusion

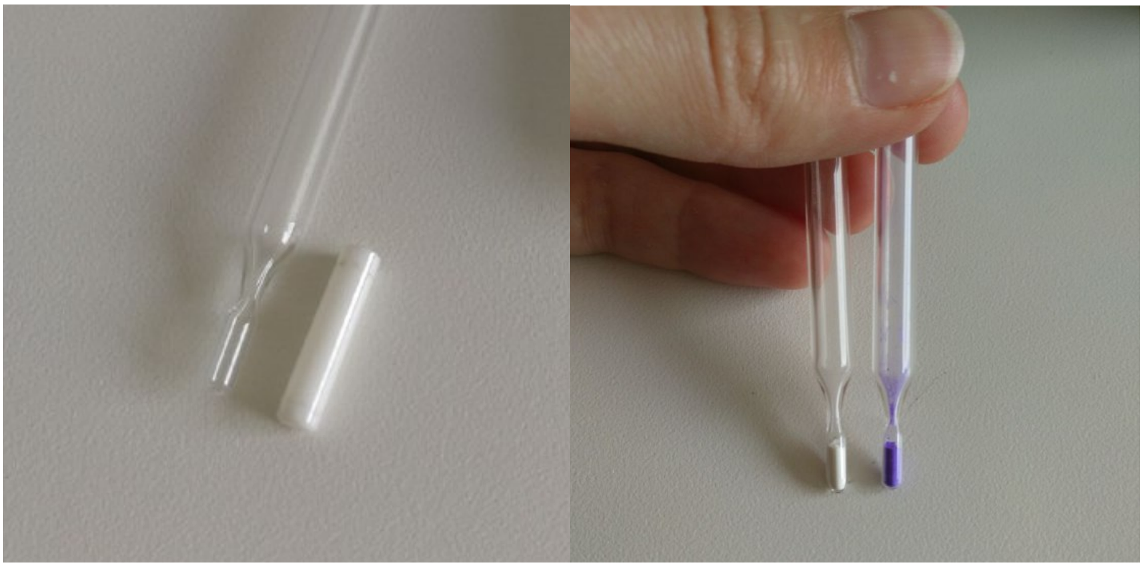

Fig. S7: Glass tubes for MAS-NMR measurement under air exclusion. Left: empty glass tube next to 4 mm rotor. Right: glass tubes filled with powder samples (before vacuum and sealing), ZIF-8 (white sample), ZIF-67 (purple sample).

MAS-NMR spectra of ZIFs

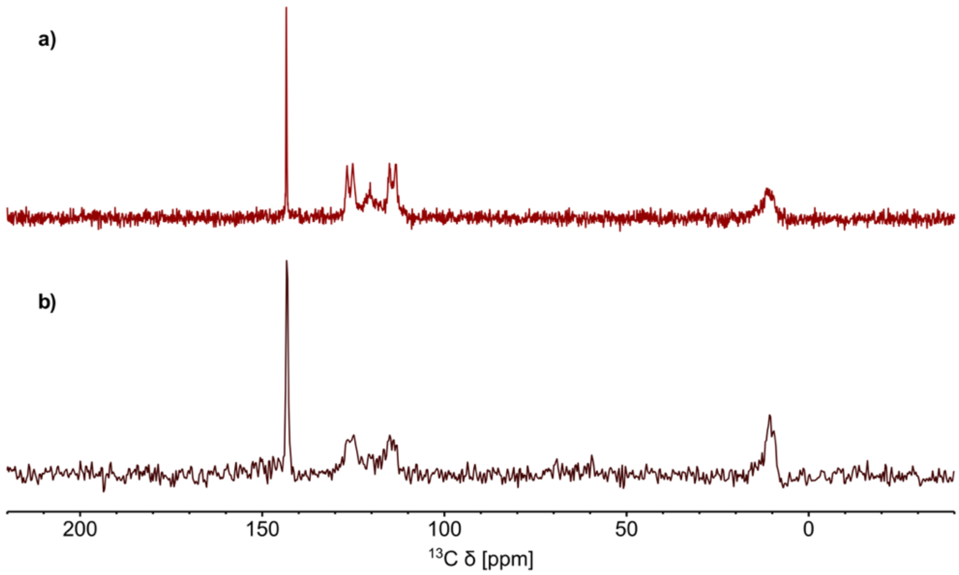

Fig. S8: a) Spectrum of ZIF-8 measured in glass tube inside rotor. Hahn echo pulse sequence, non-decoupled, 8 kHz MAS frequency, 10.000 scans.
b) Spectrum of ZIF-67 measured with regular packing method.
Hahn echo pulse sequence, non-decoupled, 8 kHz MAS frequency, 1.000 scans.

XRD spectra

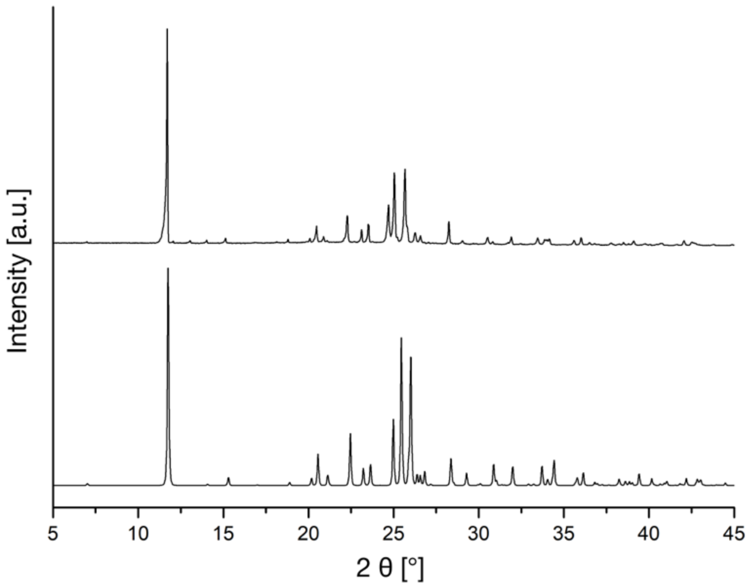

Fig. S9: XRD spectra of 2,5-dimethyl-1,3-dinitrobenzene (**8**, AYOYAP). Top: experimental data , bottom: simulated spectrum from crystal structure data (Johnston and Crather, 2011).

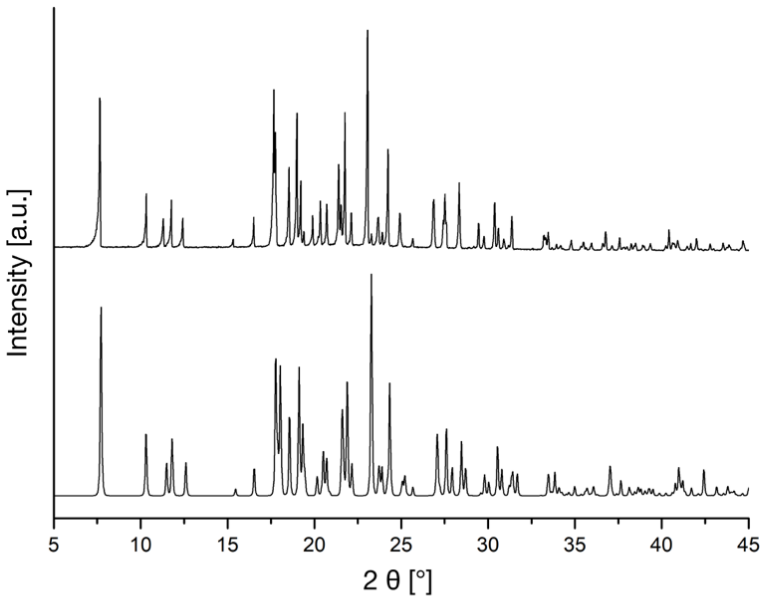

Fig. S10: XRD spectra of *N'*-(3,4-difluorobenzylidene)-4-methylbenzenesulfonohydrazide (**12**, NUQDUA). Top: experimental data , bottom: simulated spectrum from crystal structure data (Wang and Yan, 2015).

Tunnel frequency fit

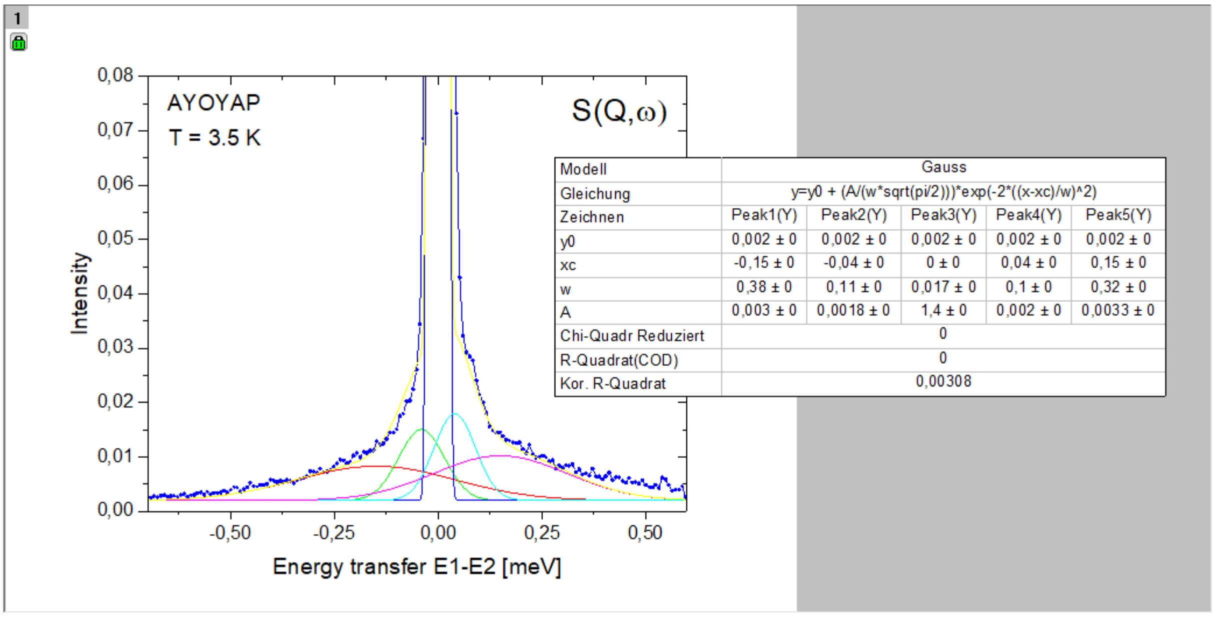

Fig. S11: Origin Fit for 2,5-dimethyl-1,3-dinitrobenzene (**8**, AYOYAP).

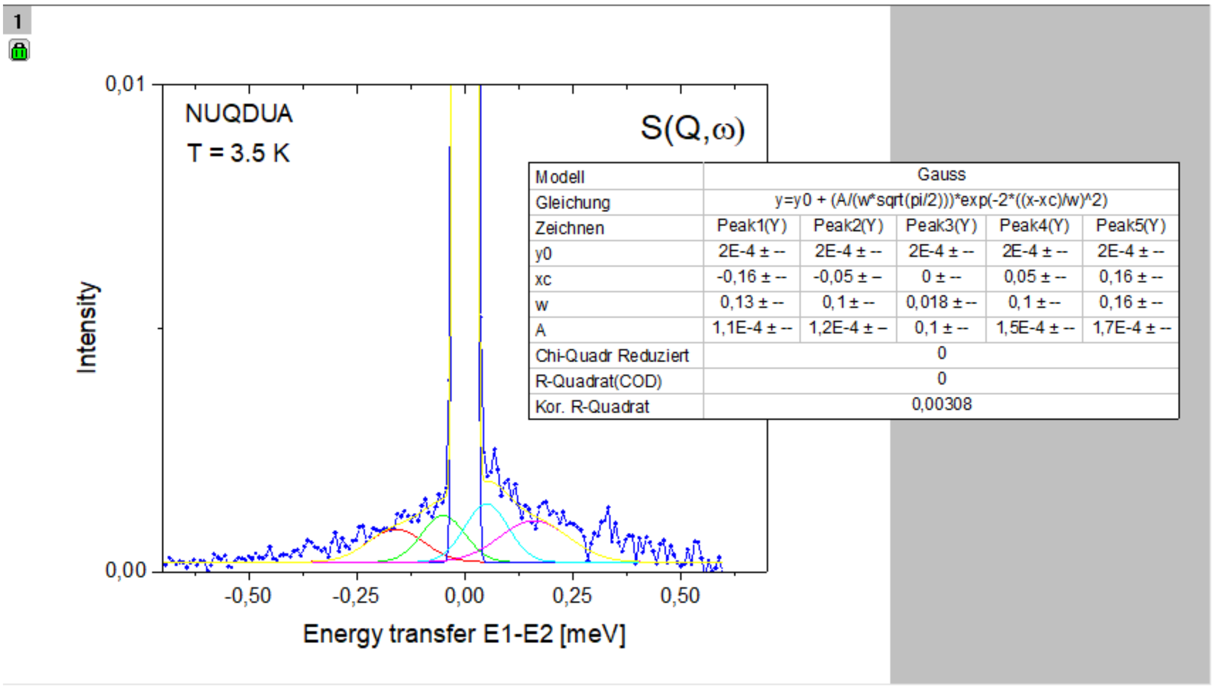

Fig. S12: Origin Fit for *N′*-(3,4-difluorobenzylidene)-4-methylbenzenesulfonohydrazide (**12**, NUQDUA).

Literature of Supplementary

[1] Faber, A., Lemke, A., Spangenberg, B. & Bolte, M. Three hydrohalogenides of organic nitrogen bases. *Acta Crystallogr. Sect. C Cryst. Struct. Commun.* **55**, IUC9900156 (1999).

[2] Arputharaj, D. S., Hathwar, V. R., Guru Row, T. N. & Kumaradhas, P. Topological Electron Density Analysis and Electrostatic Properties of Aspirin: An Experimental and Theoretical Study. *Cryst. Growth Des.* **12**, 4357–4366 (2012).

[3] Bois, C. Structure du m -crésol. *Acta Crystallogr. Sect. B Struct. Crystallogr. Cryst. Chem.* **29**, 1011–1017 (1973).

[4] Hernandez, O., Cousson, A., Plazanet, M., Nierlich, M. & Meinnel, J. The low-temperature phase of 1,3-dibromo-2,4,6-trimethylbenzene: a single-crystal neutron diffraction study at 120 and 14 K. *Acta Crystallogr. Sect. C Cryst. Struct. Commun.* **59**, o445–o450 (2003).
